# Challenges in modelling the proportion of undiagnosed HIV infections in Sweden

**DOI:** 10.2807/1560-7917.ES.2019.24.14.1800203

**Published:** 2019-04-04

**Authors:** Emmi Andersson, Fumiyo Nakagawa, Ard van Sighem, Maria Axelsson, Andrew N Phillips, Anders Sönnerborg, Jan Albert

**Affiliations:** 1 Division of Clinical Microbiology, Department of Laboratory Medicine, Karolinska Institute, Stockholm, Sweden; 2 Department of Clinical Microbiology, Karolinska University Hospital, Stockholm, Sweden; 3 Institute for Global Health, University College London, London, United Kingdom; 4 Stichting HIV Monitoring, Amsterdam, the Netherlands; 5 Department of Public Health Analysis and Data Management, Public Health Agency of Sweden, Solna, Sweden; 6 Department of Infectious Diseases, Karolinska University Hospital, Stockholm, Sweden; 7 Unit of Infectious Diseases, Department of Medicine Huddinge, Karolinska Institute, Stockholm, Sweden; 8 Department of Microbiology, Tumor and Cell Biology, Karolinska Institute, Stockholm, Sweden

**Keywords:** HIV infection, epidemiology, migration, men who have sex with men, MSM, sexually transmitted infections

## Abstract

**Background:**

Sweden has a low HIV prevalence. However, among new HIV diagnoses in 2016, the proportion of late presenters and migrants was high (59% and 81%, respectively). This poses challenges in estimating the proportion of undiagnosed persons living with HIV (PLHIV).

**Aim:**

To estimate the proportion of undiagnosed PLHIV in Sweden comparing two models with different demands on data availability and modelling expertise.

**Methods:**

An individual-based stochastic simulation model of HIV positive populations (SSOPHIE) and the incidence method of the European Centre for Disease Prevention and Control (ECDC) HIV Modelling Tool were applied to clinical, surveillance and migration data from Sweden 1980–2016.

**Results:**

SSOPHIE estimated that the proportion of undiagnosed PLHIV in 2013 was 26% (n = 2,100; 90% plausibility range (PR): 900–5,000) for all PLHIV, 17% (n = 600; 90% PR: 100–2,000) for men who have sex with men (MSM), 35% in male (n = 300; 90% PR: 200–700) and 34% in female (n = 400; 90% PR: 200–800) migrants from sub-Saharan Africa (SSA). The estimates for the ECDC model in 2013 were 21% (n = 2,013; 95% confidence interval (CI): 1,831–2,189) for all PLHIV, 15% (n = 369; 95% CI: 299–434) for MSM and 21% (n = 530; 95% CI: 436–632) for migrants from SSA.

**Conclusions:**

The proportion of undiagnosed PLHIV in Sweden is uncertain. SSOPHIE estimates had wide PR. The ECDC model estimates were unreliable because migration was not accounted for. Better migration data and estimation methods are required to obtain reliable estimates of proportions of undiagnosed PLHIV in similar settings.

## Introduction

Estimating the number of undiagnosed persons living with HIV (PLHIV) is crucial to describe the HIV care continuum and to address the Joint United Nations Programme on HIV/AIDS (UNAIDS) 90–90–90 target [1]. This requires reliable data on the number of undiagnosed and diagnosed PLHIV, the proportion of diagnosed PLHIV receiving antiretroviral therapy (ART) and the proportion of those receiving ART who are virally suppressed [1]. Unlike the other parameters in the HIV care continuum, the undiagnosed proportion of PLHIV cannot be directly monitored and must be estimated.

Sweden has a low prevalence of diagnosed PLHIV (0.07% (data not shown) compared to 0.4% in adults in the European World Health Organization (WHO) Region [2]) and a low incidence of new HIV diagnoses, with 430 new cases reported within the national surveillance programme in 2016. The number of new cases with reported transmission within Sweden has decreased and has remained below 100 cases per year between 2013 and 2017 [3]. Thus, most HIV infections diagnosed in Sweden are reported to have been acquired abroad. A large proportion of new HIV diagnoses in Sweden occur in migrants with 81% in 2016 (77%, 72%, 80%, 84% and 80% between 2011 and 2015, respectively) according to data from the Public Health Agency of Sweden (data not shown). Migrants from sub-Saharan Africa (SSA) constitute the largest group (38% of all new infections in 2016) and most of them are reported to have acquired their HIV infection in their country of origin. However, a recent study indicates that doctors underestimated the occurrence of transmissions after arrival to Sweden. Thus, a CD4+ T-cell decline trajectory model applied to migrants diagnosed with HIV in Sweden 1983–2013 estimated that 19% of migrants were infected post-migration, rather than 12% as officially reported [4]. Furthermore, between 2009 and 2012, 58% of individuals diagnosed with HIV in Sweden were late presenters [5], as defined by a CD4 + T-lymphocyte (CD4+) count < 350 cells/µl at diagnosis [6]. Late presentation was associated with origin in SSA, eastern Europe, Asia and the Pacific region [5]. Data from the National Quality Registry for HIV (InfCareHIV) [7] Sweden shows that late presentation still is common (59% in 2016) (data not shown).

In Sweden, most parameters of the HIV care continuum are well documented in InfCareHIV, which has had national coverage since 2008. Sweden was reported to be the first country to achieve the UNAIDS 90–90–90 target [8] but the estimate of the proportion of undiagnosed PLHIV is uncertain; as in many other European countries [9]. The proportion of undiagnosed PLHIV in Sweden was estimated to be 12–20% in 2006 by Hamers et al*.* [10] but no estimates have been published since.

Access to high quality surveillance data combined with near complete coverage of diagnosed individuals with HIV in InfCareHIV since 2008, as well as good coverage of entry of CD4+ cell counts at diagnosis (95% in 2013), country of origin (99% in 2013) and transmission route (96% in 2013) are good starting points for estimating undiagnosed individuals with HIV in Sweden.

We evaluated two models with different requirements on data availability and modelling expertise to estimate the proportion of undiagnosed PLHIV in Sweden. Migration may bias estimates of undiagnosed HIV infections, because HIV-positive migrants should only be counted as undiagnosed in Sweden after their arrival to the country. The individual based stochastic simulation model of HIV positive populations (SSOPHIE) [11-13] specifically accounts for both in-country infections and infections likely to have occurred abroad, but it is labour and computing intensive. The incidence method in the HIV Modelling Tool provided by the European Centre for Disease Prevention and Control (ECDC) [14,15] was developed to use routinely collected HIV surveillance data but does not take migration into account. We wanted to compare these available models to see how they performed on data from the Swedish HIV epidemic. Both models produce estimates of several stages of the HIV care continuum but in this analysis, we only focus on estimating the proportion of undiagnosed PLHIV in Sweden.

## Methods

### Data sources

InfCareHIV Sweden is a national quality assurance registry, clinical decision tool and research database. It has full national coverage since 2008 and covers the major cities including Stockholm, Gothenburg and Malmö since the 1980s. Clinical, epidemiological and laboratory data on all registered patients with an HIV diagnosis in Sweden are entered and a well-structured system for quality control ensures data reliability [4,16]. As at 1 June 2014, the date of data extraction used for the SSOPHIE modelling, 9,647 patients were registered in InfCareHIV Sweden. Complementary data extraction for the ECDC Modelling Tool was carried out on 13 March 2017 at which point 10,858 patients were registered. This extraction consisted of data for the years 2014–16 and HIV diagnoses within CD4+ count strata as explained in Table 1. Only accumulated data on patient populations were extracted and no patient identifiers were used.

**Table 1:** Data items and sources to inform the ECDC HIV Modelling Tool

Data item (per year)	Data source 1	Year	Data source 2	Year
Number of HIV-1 diagnoses	Public Health Agency of Sweden	1984–1995	InfCareHIV	1996–2016
Number of AIDS diagnoses	Public Health Agency of Sweden	1983–2008	InfCareHIV	2009–2016
Number of simultaneous HIV/AIDS diagnoses	Public Health Agency of Sweden	1983–2002	InfCareHIV	2003–2016
Number of deaths in HIV-1 infected^a^	Public Health Agency of Sweden	1983–1994	InfCareHIV	1995–2016
Number of HIV-1 diagnoses with CD4+ ≥ 500 cells/μl and no concurrent AIDS diagnosis	InfCareHIV	1983–2016		
Number of HIV diagnoses with CD4+ 350–499 cells/μl and no concurrent AIDS diagnosis	InfCareHIV	1983–2016		
Number of HIV diagnoses with CD4+ 200–349 cells/μl and no concurrent AIDS diagnosis	InfCareHIV	1983–2016		
Number of HIV diagnoses with CD4+ < 200 cells/μl and no concurrent AIDS diagnosis	InfCareHIV	1983–2016		

AIDS: acquired immunodeficiency syndrome; ECDC: European Centre for Disease Prevention and Control; InfCareHIV: National Quality Registry for HIV.

^a^For heterosexual migrants from sub-Sahara, data source 1 covered 1988–2006 and data source 2 covered 2007–13.

Since 1985, all HIV cases in Sweden are anonymously reported to the Public Health Agency of Sweden by the clinical doctor and the clinical laboratory that confirmed HIV under the Communicable Diseases Act [17]. From 1983 until 2005 reporting of acquired immunodeficiency syndrome (AIDS) was mandatory. Records of the number of HIV diagnoses, AIDS diagnoses and deaths per year within each transmission group were extracted from 1980 until 2013.

Data on general migration from SSA were used in SSOPHIE. These data were based on the number of permits to stay in Sweden granted to persons born in SSA annually 1980–2014 as recorded by Statistics Sweden ([18] and personal communication Lo Mildh, Statistics Sweden, 30 October 2015). These data were used because both the InfCareHIV database and the Public Health Agency of Sweden have incomplete records regarding when foreign-born HIV-positive persons first arrived in Sweden.

HIV type 2 represents < 0.5% of all HIV infections in Sweden, due to this we focused on HIV type 1, hereafter referred to as HIV.

### Ethical approval

Ethical permit 532–11 with amendment T996–11 from the Regional Ethical Review Board in Gothenburg covers this study.

### Data items and model populations

The data used by the models to inform estimates of undiagnosed infection were obtained from InfCareHIV database and the Public Health Agency of Sweden. For each data item, the coverage and data quality for the two sources was assessed over the study period and the source with the best coverage was chosen. For case reporting national surveillance data was used from the beginning of the epidemic until the mid-1990s and InfCareHIV data thereafter. CD4+ cell counts at diagnosis were extracted from InfCareHIV. A slight under-reporting of AIDS cases is assumed after 2005 and compensated for by 15% in SSOPHIE. The data items and their sources are presented in Table 2 for SSOPHIE and Table 1 for the ECDC Modelling Tool.

**Table 2:** Data items and sources to inform SSOPHIE

Data item (per year)	Data source 1	Year	Data source 2	Year
Number of HIV-1 diagnoses^a^	Public Health Agency of Sweden	1980–1995	InfCareHIV	1996–2013
Number of AIDS diagnoses^b^	Public Health Agency of Sweden	1980–2008	InfCareHIV	2009–2013
Number of simultaneous HIV/AIDS diagnoses^a^	Public Health Agency of Sweden	1980–2002	InfCareHIV	2003–2013
Number of deaths in HIV-1 infected^c^	Public Health Agency of Sweden	1983–1994	InfCareHIV	1995–2013
Median CD4+-count at HIV-1 diagnosis	InfCareHIV	1983–2013		
Number of people seen for HIV-1 care	InfCareHIV	1983–2013		
Number of people receiving ART	InfCareHIV	1987–2013		
Percent of people diagnosed late (CD4+ < 200 cells/μl)^d^	InfCareHIV	1983–2013		
Percent of people diagnosed promptly (CD4+ ≥ 350 cells/μl)^d^	InfCareHIV	1983–2013		
Percent of people on ART with CD4+ ≥ 350 cells/μl^d^	InfCareHIV	1987–2013		
Percent of people on ART with viral load < 500 copies/ml	InfCareHIV	1996–2013		
Death rates in the general population	The National Board of Health and Welfare	2008		
Proportion of HIV-1 infected with HCV/HBV	InfCareHIV	2014		
Linkage to care	InfCareHIV	2013		
Retention in care	InfCareHIV	2013		
Proportion with viral suppression	InfCareHIV	2013		

AIDS: acquired immunodeficiency syndrome; ART: antiretroviral therapy; HBV: hepatitis B virus; HCV: hepatitis C virus; InfCareHIV: National Quality Registry for HIV; SSOPHIE: Stochastic Simulation model of Outcomes of People with HIV In Europe.

^a^ Surveillance reports for AIDS from 1980–84 were reregistered after availability of HIV testing in 1985.

^b^ Possible under-reporting after 2005 is compensated by factoring in 15% in the model.

^c^ For heterosexual migrants from sub-Sahara, data source 1 covered 1988–2006 and data source 2 covered 2007–13.

^d^ CD4+ cell counts in these formats were used only for the whole population.

The continuum of HIV care was monitored using data from the InfCareHIV database; figures on linkage to care, retention in care and retention on ART from 2013 was > 99%, > 99% and > 98% respectively. Of patients retained in care, 98.5% started ART within a year if eligible according to the national treatment guidelines at that time, 94% of persons on ART had viral loads < 50 copies/ml (Veronica Svedhem, personal communication, 22 December 2014).

For both models, estimates for the undiagnosed proportion were made for all PLHIV as well as separately for the following transmission groups: men having sex with men (MSM), heterosexually infected migrants from SSA and other heterosexually infected patients, i.e. Swedish-born persons and migrants from other areas in the world than SSA. The number of individuals in the subpopulations do not add up to the total number of PLHIV, because not all PLHIV are part of one of these three subgroups. People with injecting drug use and heterosexually infected migrants from south-east Asia are also relevant transmission groups in Sweden, but they were too small to model individually.

### SSOPHIE

SSOPHIE was developed by Nakagawa et al. within the Stochastic Simulation of Outcomes of People with HIV In Europe project in EuroCoord (EuroCoord-SSOPHIE project) [11-13]. Briefly, the model accounts for domestic and non-domestic infections and uses clinical and observational data to simulate hypothetical HIV-infected individuals from the beginning of a specified HIV epidemic until the present time. Approximate Bayesian computation (ABC) methods are used to calibrate the simulated data to the observed data. The model was developed for settings where extensive surveillance information is available but is flexible with respect to which parameters are required and depending on data reliability, parameters can be weighted and/or fixed. To account for suggested epidemiological differences in access to care, diagnosis rate etc. transmission groups can be categorised in order to calibrate and obtain estimates for the entire population as well as separately for larger subgroups.

Estimates on migration from SSA were included in SSOPHIE. We used the approach described by Nakagawa et al. [13] Briefly, we simulated a ‘pool’ of HIV-positive people living in SSA using Spectrum/Estimation and Projection Package (EPP) and an HIV-incidence curve based on UNAIDS estimates [19,20]. From this pool, we assumed a rate of migration of HIV-infected persons based on the national data on migration from SSA to Sweden, under the assumption that HIV-infected and uninfected persons had a similar rate of migration. This enabled us to make estimates relating to the transmission subgroup of heterosexually infected migrants from SSA.

SSOPHIE was run on data until 2013 for all PLHIV and the subgroups. For the heterosexual transmission groups, estimates were produced separately for men and women. SSOPHIE estimates are given as point estimates with 90% plausibility ranges (PR) that indicate the certainty of the point estimate.

A total of 30,000 simulations were performed, of which 16,060 were not terminated prematurely; 748 runs were under the calibration-score < 0.3 (i.e. they resulted in outputs that were quite close to the observed data with an average deviation from the observed data across all data items < 30%). We aimed for a calibration-score as low as possible given that we were attempting to reconcile data from several sources on various aspects of the epidemic. We balanced the aim for a low calibration-score with the desire to have at least 100 parameter sets so that we could consider a range of ways in which the epidemic could be reconstructed. Too few runs (only 17) were under the desired threshold of calibration-score < 0.25 and the results were therefore based on the 107 parameter sets with calibration-score < 0.27. Data items used for calibration are shown in Supplementary Table S1 and examples in Supplementary Figure S1.

### The ECDC HIV Modelling Tool

The incidence method in the ECDC HIV Modelling Tool version 1.3.0 is a multi-state back-calculation model based on maximum likelihood statistics [14,15]. The method first estimates HIV incidence over time and time from infection to diagnosis by CD4+ count strata and then estimates the size of the HIV-positive population. Migration is not addressed in the model but subgroups can be analysed separately.

The model included data from 1983 up to 2016 for all PLHIV and separately for the subgroups MSM, heterosexual migrants from SSA and other heterosexually infected individuals. An optimised model, as assessed by the goodness of fit results, was chosen and the same settings were used for all subgroups. HIV diagnoses were modelled from 1985, but the model was not fitted to data for 1985 and 1986 to avoid the peak of diagnoses reflecting the introduction of HIV-testing. Diagnosis rate was differentiated by CD4+ count categories from 1987 and improvements in fit were obtained by increasing the number of knots in the incidence curve to six and adjusting the length of time intervals (Supplementary Table S2a). Bootstrap analysis (1,000 iterations) was applied to produce 95% confidence intervals (CI) of the estimates from the final model.

## Results

### SSOPHIE estimates

**Figure 1:**
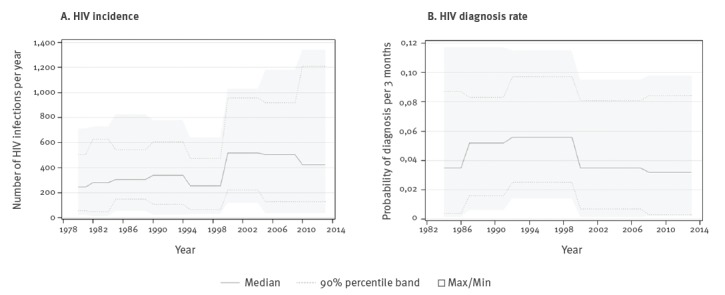
HIV (A) incidence of domestic infections and (B) diagnosis rate in PLHIV, by year, Sweden, 1980–2013

Using SSOPHIE, we modelled HIV incidence and HIV diagnosis rate to estimate the proportion of undiagnosed PLHIV. Figure 1a shows that SSOPHIE estimated a median incidence of 400–500 new domestic HIV infections per year during 2000–13 with an increasing 90% PR. Figure 1b shows that the diagnosis rate was estimated to have decreased from the end of the 90s until 2013.

PLHIV: persons living with HIV.

**Figure 2:**
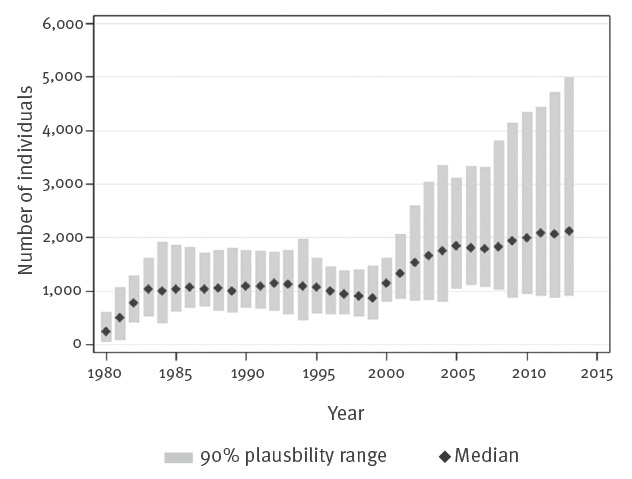
Estimated number of persons living with undiagnosed HIV, by year, Sweden, 1980–2013

The number of undiagnosed individuals among all PLHIV during 1980–2013 as estimated by SSOPHIE is shown in Figure 2 and the estimates for subgroups are summarised in Table 3. The proportion of undiagnosed PLHIV was 26% in 2013 (n = 2,100; 90% PR: 900–5,000). The estimate of undiagnosed infections was lowest in MSM; 17% (n = 600; 90% PR: 100–2,000), and highest in heterosexual men from sub-Saharan Africa; 35% (n = 300; 90% PR: 200–700). The proportion undiagnosed among heterosexually infected persons, excluding migrants from SSA, was 30% in both men (n = 300; 90% PR: 100–1,000) and women (n = 400; 90% PR: 100–1,200).

**Table 3:** Proportion and number of undiagnosed PLHIV, as estimated by SSOPHIE and the ECDC HIV Modelling Tool, Sweden, 2013 and 2016

Transmission group	SSOPHIE 2013		ECDC model 2013		ECDC model 2016	
	Proportion	n (90% PR)	Proportion (95% CI)	n (95% CI)	Proportion (95% CI)	n (95% CI)
All PLHIV^a^	26%	2,100 (900–5,000)	21% (20–23%)	2,013 (1,831–2,189)	20% (17–23%)	2,107 (1,688–2,577)
MSM	17%	600 (100–2,000)	15% (12–17%)	369 (299–434)	17% (13–22%)	518 (355–706)
Migrants from SSA			21% (18–24%)	530 (436–632)	19% (14–26%)	535 (359–789)
Male	35%	300 (200–700)				
Female	34%	400 (200–800)				
Other heterosexual			21% (18–25%)	502 (407–600)	22% (16–30%)	589 (398–884)
Male	30%	300 (100–1,000)				
Female	30%	400 (100–1,200)				

CI: confidence interval; ECDC: European Centre for Disease Prevention and Control; MSM: men having sex with men; PLHIV: persons living with HIV (diagnosed or undiagnosed); PR: plausibility range; SSA: sub-Saharan Africa; SSOPHIE: Stochastic Simulation model of Outcomes of People with HIV In Europe.

^a^ The number of individuals in the subpopulations do not add up to the total number of PLHIV, because not all PLHIV are part of one of these three subgroups.

### The ECDC HIV Modelling Tool estimates

**Figure 3:**
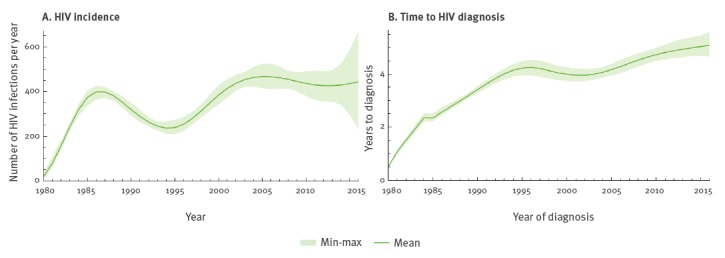
HIV (A) incidence and (B) time to diagnosis, by year, Sweden, 1980–2016

The estimates of the HIV incidence and time to diagnosis obtained with the ECDC HIV Modelling Tool are presented in Figure 3. The incidence of HIV infections was estimated to have stabilised between 400 and 500 new infections per year since 2001 (Figure 3a). However, the ECDC model does not account for migration, this means that infection could have occurred abroad and those foreign-born persons could have been living with HIV abroad before being diagnosed in Sweden. The time from infection to diagnosis was estimated to have gradually increased from under 3 years in the 1980s to 5 years in 2016 (Figure 3b). Time to diagnosis was highest among migrants from SSA, over 6 years since 2011 and lowest in MSM, 3 to 4 years since 2003 (data not shown).

**Figure 4:**
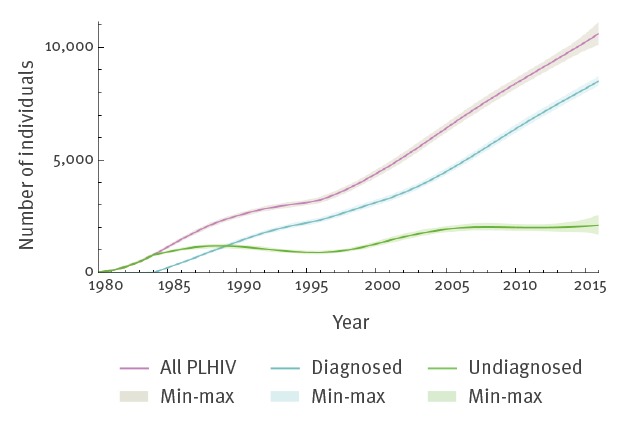
Number of diagnosed and undiagnosed persons living with HIV, by year, Sweden, 1980–2016

The ECDC model estimates of the proportion of undiagnosed PLHIV in 2013 are shown in Figure 4 and Table 3. The overall estimate was 21% (n = 2,013; 95% CI: 1,831–2,189), with 15% (n = 369; 95% CI: 299–434) in MSM, 21% (n = 530; 95% CI: 436–632) in migrants from SSA, and 21% (n = 502; 95% CI: 407–600) in other heterosexually infected persons. For the ECDC model, input data until 2016 were available and used to obtain estimated proportions of undiagnosed PLHIV (Table 3). The estimate for all PLHIV was 20% (n = 2,107; 95% CI: 1,688–2,577), in MSM 17% (n = 518; 95% CI: 355–706), in migrants from SSA 19% (n = 535; 95% CI: 359–789) and in other heterosexually infected persons 22% (n = 589; 95% CI: 398–884).

PLHIV: persons living with HIV.

## Discussion

We found that the estimated proportion of undiagnosed PLHIV in Sweden in 2013 was comparable between SSOPHIE (26%) and the ECDC model (21%). The ECDC model suggested that the proportion of undiagnosed was similar in 2016 (20%). Both methods estimated around 2,000 individuals living with undiagnosed HIV in Sweden. However, the SSOPHIE estimate had a wide PR and the ECDC model did not account for migration, which makes the accuracy of both estimates uncertain.

The domestic incidence of HIV infections in Sweden estimated with SSOPHIE (400–500 infections per year during 2000–13) seems unrealistically high given that a total of 400–500 new HIV cases (domestic and non-domestic) per year were reported to the Public Health Agency of Sweden during the same time period of whom a majority (76% in 2013) were reported to be non-domestic [3]. The ECDC model had narrow 95% CI when considering the wide PR from SSOPHIE, but did not adjust for infections acquired abroad and therefore did not take into account that migrant PLHIV only contribute to the undiagnosed fraction after arrival to Sweden. Consequently, the SSOPHIE and the ECDC point estimates should be interpreted with caution.

Based on the preconception regarding the proportion of undiagnosed PLHIV among Swedish epidemiologists and clinicians, both estimates are higher than expected. The estimates also exceed the 12–20% undiagnosed in 2006 estimated by Hamers et al. utilising surveillance data from 2005 and the HIV Synthesis model (a predecessor of SSOPHIE) [10]. In our study, both models estimated around 30% undiagnosed infections in 2006 (data not shown). Neither are the results in line with an estimate of 10.8% in 2015 – based on advanced modelling on multiple-biomarker estimates of time between infection and diagnosis [21].

The point estimates for undiagnosed infections in male (35%) and female (34%) migrants from SSA and in other individuals with heterosexual transmission (30%) were higher in SSOPHIE, but the PRs of SSOPHIE overlapped with the 95% CIs of the ECDC estimates, indicating that there was no true discrepancy between the results.

Both models predicted that MSM have the lowest proportion of undiagnosed infections. Historically, this subpopulation has been less influenced by migration making it more suitable for modelling using these two methods. However, in 2016, 74% of MSM diagnosed with HIV in Sweden were reported to have been infected abroad and 71% were born in another country (data not shown), suggesting that migration is of increasing importance also in this group.

Our surveillance data are of high quality, but had some limitations; CD4+ cell count medians from the 1980s might be slightly biased by incomplete coverage but this is unlikely to have affected our results. The ECDC HIV Modelling Tool takes missing data into account by assuming that CD4+ count data are missing at random in individuals with no concurrent AIDS diagnosis. We expect some under-reporting of AIDS after 2005 when reporting was no longer mandatory. In SSOPHIE, we have compensated for this, but in the ECDC model, missing data on concurrent HIV/AIDS diagnoses could potentially lead to estimates of time from HIV infection that are shorter than the true values.

There are several limitations in estimating the proportion of undiagnosed PLHIV in Sweden using these two models. First, it is difficult to enumerate a ‘hidden’ population on whom we have no direct data. Second, the models were applied to small numbers with the Swedish HIV epidemic limited to 400–500 new reported cases per year and even smaller numbers for each modelled subgroup. Third, how to properly account for migration, as migrants from SSA and other areas of the world constitute a large proportion of new HIV cases in Sweden. SSOPHIE explicitly attempts to account for migration, but as our migration data were imprecise the HIV prevalence in SSA and the rate of migration from this region to Sweden was used in the model; weaknesses in incomplete migration data from Sweden and any biases introduced by the assumptions on migration of PLHIV from SSA might have affected the results. Also, migration was only considered in the subgroup heterosexually infected migrants from SSA, even though some migrants have other transmission routes and origin. The ECDC model does not account for migration at all. Fourth, the characteristics of the Swedish HIV epidemic have changed substantially since the 1980s, with migration becoming an increasingly important factor. Thus, the calibration to historical data in both models may not be have been an optimal approach in Sweden. The feasibility of national estimates of undiagnosed infections in a country where both travel and migration have a large impact on the HIV epidemic is dependent on the inclusion of detailed travel and migration data in surveillance reporting as well as the availability of updated and complete national migration data. Modelling approaches for settings with similar patterns of HIV epidemics need to consider migration as an important factor. Since no method will be perfect, comparisons of results from different approaches while understanding their pros and cons are important.

In conclusion, there remains uncertainty over the proportion of undiagnosed PLHIV in Sweden and both methods used have limitations. Consequently, it is difficult to properly evaluate the HIV care continuum in the country. The main challenges with estimating the proportion of undiagnosed PLHIV in Sweden are the high proportion of foreign-born PLHIV, a comparably small number of new cases per year and heterogeneity between subgroups of PLHIV. A combination of better input data on migration and updated or new models are likely required to address these challenges.

## Supplementary Data

476000 Supplementary Figures S1

## Supplementary Data

15000 Supplementary Tables S1
