# Supplementary material for: Challenges in modelling the proportion of undiagnosed HIV infections in Sweden
Source: Euro Surveill. 2019 Apr 4;24(14):1800203. doi: 10.2807/1560-7917.ES.2019.24.14.1800203 (PMC6462786; doi:10.2807/1560-7917.ES.2019.24.14.1800203)

**Supplementary Figures 1 and 2**

**This supplementary material is hosted by Eurosurveillance as supporting information alongside the article *“Challenges in modelling the proportion of undiagnosed HIV infections in Sweden”* on behalf of the authors who remain responsible for the accuracy and appropriateness of the content. The same standards for ethics, copyright, attributions and permissions as for the article apply. Eurosurveillance is not responsible for the maintenance of any links or email addresses provided therein.**

**Supplementary figure 1.**

Examples of calibration of SSOPHIE to observed data.

Solid diamonds are used for calibration.

Supplementary figure 1a.


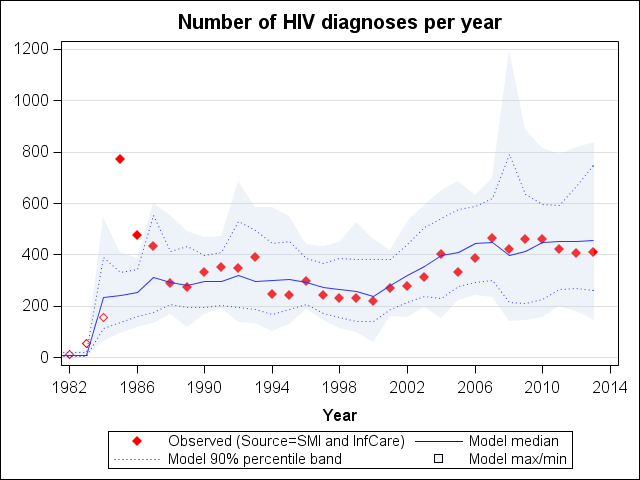


Supplementary figure 1b.


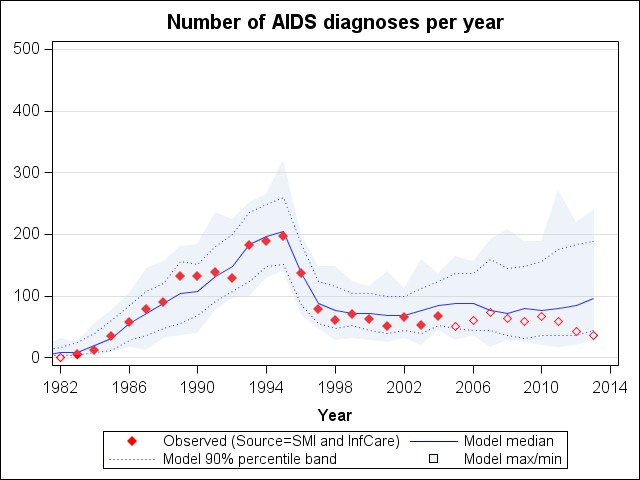


Supplementary figure 1c.


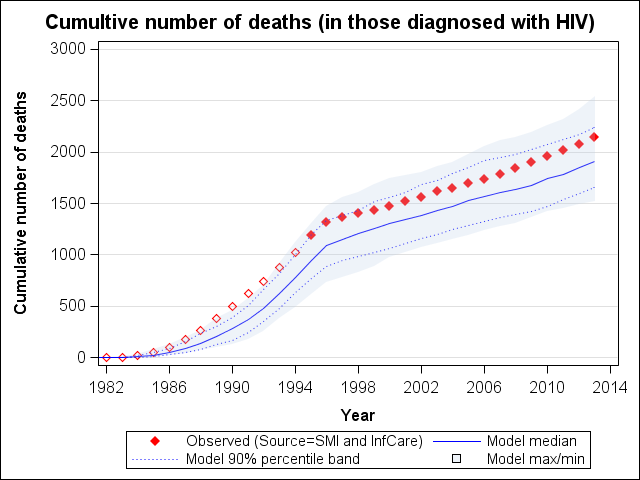


Supplementary figure 1d.


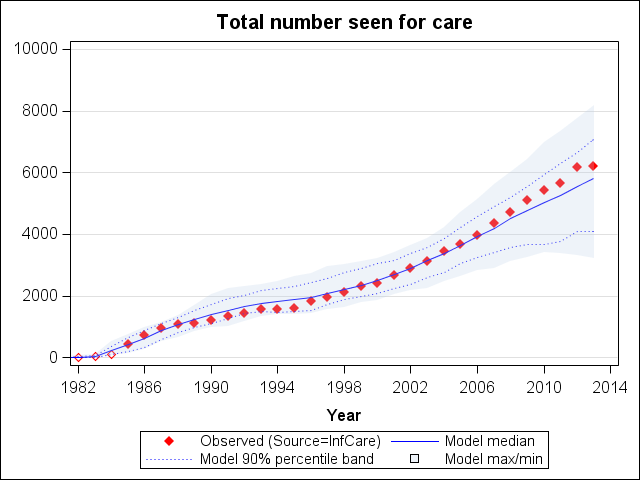


Supplementary figure 1e.


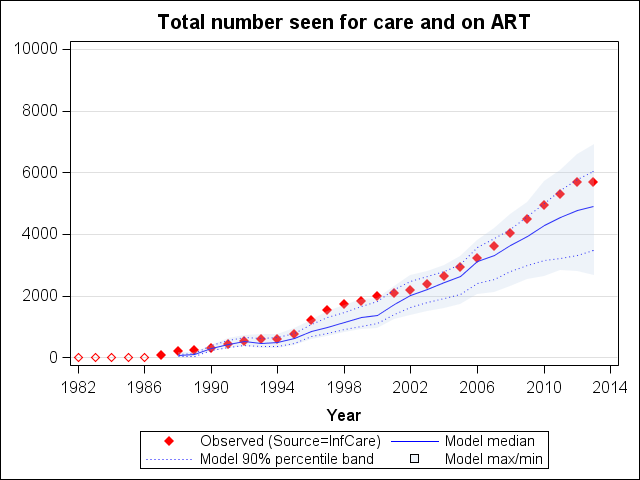


**Supplementary figure 2.**

Observed and modelled data in all PLHIV in ECDC HIV Modelling Tool

Supplementary figure 2a.

**Observed and modelled total HIV diagnoses**


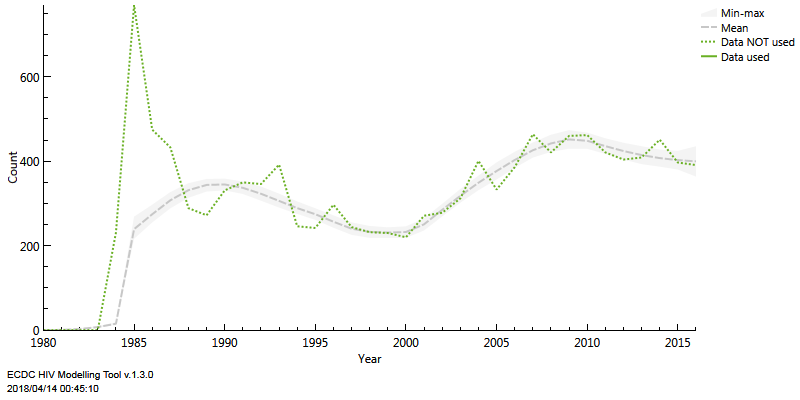


Supplementary Figure 2b.

**Observed and modelled HIV diagnoses with CD4 ≥ 500 cells/μl and no AIDS**


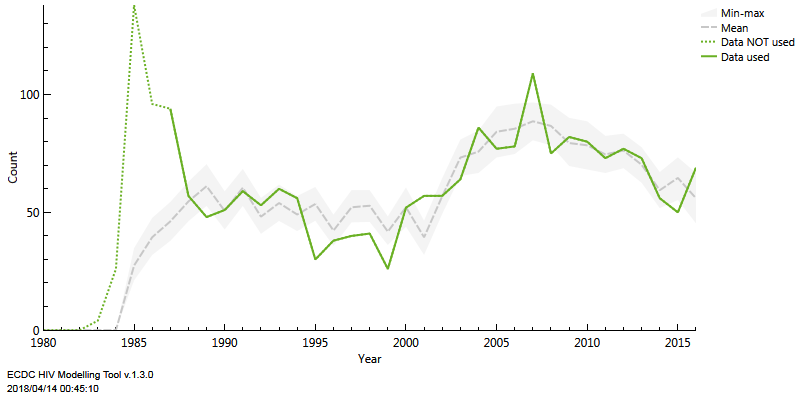


Supplementary figure 2c.

**Observed and modelled HIV diagnoses with CD4 350-499 cells/μl and no AIDS**


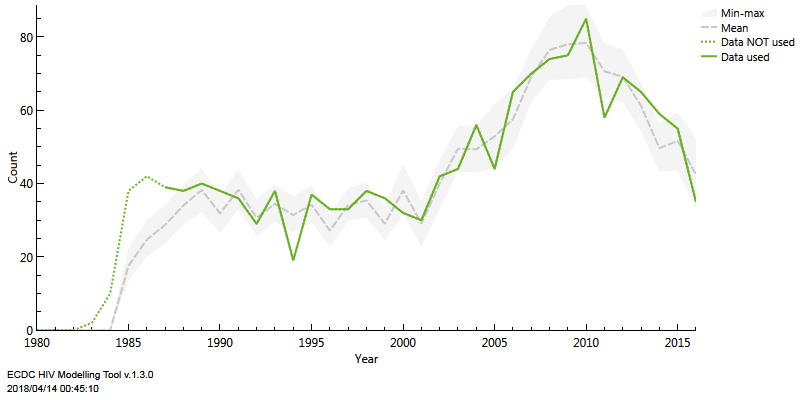


Supplementary figure 2d.

**Observed and modelled HIV diagnoses with CD4 200-349 cells/μl and no AIDS**


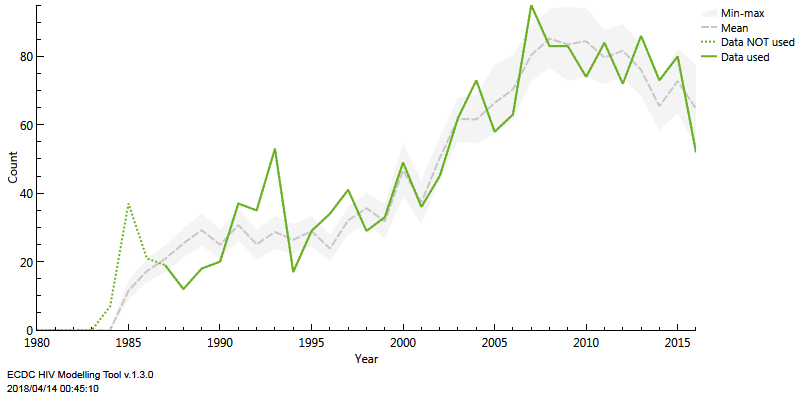


Supplementary figure 2e.

**Observed and modelled HIV diagnoses with CD4 < 200 cells/μl and no AIDS**


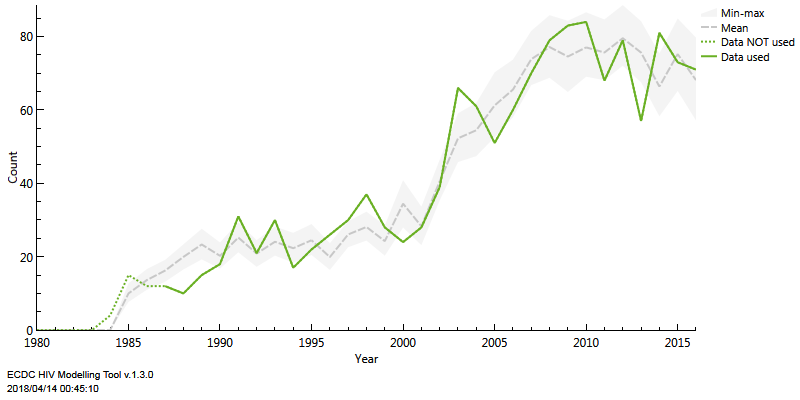


Supplementary figure 2f.

**Observed and modelled concurrent HIV/AIDS diagnoses**


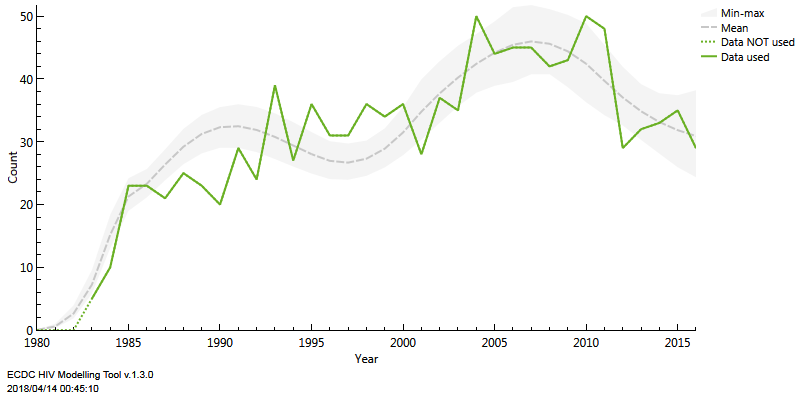


Supplementary figure 2g.

**Observed and modelled AIDS diagnoses**


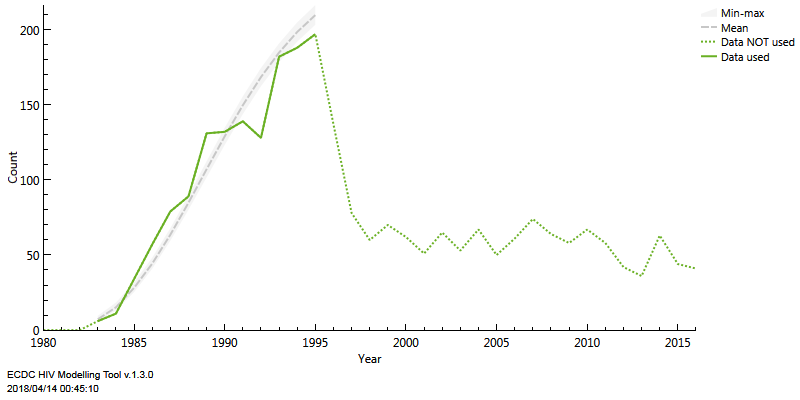

Supplement: Supplementary Figures S1 [file 1800203_ANDERSSON_Supplementary_Figures.docx]
