# Supplementary material for: Challenges in modelling the proportion of undiagnosed HIV infections in Sweden
Source: Euro Surveill. 2019 Apr 4;24(14):1800203. doi: 10.2807/1560-7917.ES.2019.24.14.1800203 (PMC6462786; doi:10.2807/1560-7917.ES.2019.24.14.1800203)
Supplement: Supplementary Tables S1 [file 1800203_ANDERSSON_Supplementary_Tables.docx]

**Supplementary Tables 1 and 2**

**This supplementary material is hosted by Eurosurveillance as supporting information alongside the article *“Challenges in modelling the proportion of undiagnosed HIV infections in Sweden”* on behalf of the authors who remain responsible for the accuracy and appropriateness of the content. The same standards for ethics, copyright, attributions and permissions as for the article apply. Eurosurveillance is not responsible for the maintenance of any links or email addresses provided therein.**

**Supplementary table 1.**

| Data used for calibration of the SSOPHIE model |
| --- |
| Number of HIV-1 diagnoses/year |
| Cumulative number of HIV-1 diagnoses per year |
| Number of AIDS-diagnoses per year |
| Cumulative number of deaths |
| Total number seen for care |
| Total number seen for care and on ART |
| Number of HIV-1 diagnoses per year in MSM |
| Number of HIV-1 diagnoses per year in migrants from SSA |
| Number of MSM seen in care |
| Number of migrants from SSA seen in care |

**Supplementary Table 2a.**

Time intervals and diagnosis rate modelling in final model in ECDC HIV Modelling Tool v.1.3.0.

| Interval | Start from new baseline | Different by CD4 count cathegories | Changing during time interval |
| --- | --- | --- | --- |
| 1980-1985 | No | No | No |
| 1985-1996 | Yes | Yes | No |
| 1996-2001 | No | Yes | Yes |
| 2001-2006 | No | Yes | Yes |
| 2006-2010 | No | Yes | Yes |
| 2010-2016 | No | Yes | Yes |

**Supplementary Table 2b.**

Goodness of fit in ECDC HIV Modelling Tool v.1.3.0.

| Population modelled | Total deviance | Observations |
| --- | --- | --- |
| All PLHIV | 284.41 | 167 |
| MSM | 187.43 | 167 |
| Migrants from SSA | 249.48 | 167 |
| Other heterosexual | 197.04 | 167 |
